# A Practical Treatise on the Special Diseases of the Skin

**Published:** 1845-07

**Authors:** 


A Practical Treatise on the Special Diseases of the Skin. By *C. M. Gibert.* Second Edition. Translated from the French by *Edgar Sheppard.* 12mo. pp. 362. Churchill, 1845.

Mr. Sheppard assigns the following reason for undertaking the translation of M. Gibert's treatise.

"It may naturally, and therefore, perhaps, not very unreasonably, be asked, Why, since there are so many works, original and translated, already published in this country, upon 'Diseases of the Skin,' I have ventured to swell their number? The plain and simple reason is this: When I was in Paris, attending the practice of M. Gibert (who has a far greater number of English students following him than any other physician or surgeon in the French metropolis,) at the Hôpital St. Louis, my ears were constantly saluted with the question 'Has M. Gibert written any work on cutaneous affections?' This being answered in the affirmative, was followed by another—'Is there any English Translation of it?' and great was the mortification of many a young aspiring dermatologist, whose knowledge of French was not very extensive, to hear a negative response."

But for the reason here assigned we should certainly have deemed the present translation uncalled for; for although, as must any work proceeding from the pen of so able and experienced a practitioner as M. Gibert, the Treatise contains much valuable matter, we have been struck during the perusal of its contents with the want of originality in its views, its far too great dependence upon the dicta of preceding writers, and the paucity of practical directions which can be called the author's own. We looked for, at the hands of the physician of so celebrated a receptacle of the diseases on which he treats, a far fuller development of his own views and practice, and a much less frequent quotation of those of authors already well known to the profession. However, we must remember he is writing for French students and practitioners; and, in this point of view, feel gratified at his frequent quotations from the English authors, and the frank acknowledgment, so rare among his countrymen, of the improvements they have introduced into the study and practice of this branch of medical science.

The first chapter of "General Considerations" is an able and useful one, and from it we will borrow some observations.

*Causes.*—The *proximate* cause of most cutaneous diseases is unknown, and has been attributed to vitiated humors, changes in the blood, or to inflammatory action, according to the nature of the dominant theory of the day.

"The wisest plan is to confine ourselves to the immediate result of observation, which has demonstrated to physicians in all ages, that the diseases of the skin are often dependent upon a special *diathesis,* which has caused, sustained, and reproduced them. The difficulty is to discriminate between the cases which are purely local, and those in which the disease is kept up by causes more or less secret, and more or less general; and on this head we ought to commend the efforts of the learned Lorry, although they have not met with the success which he expected. It is evident, for example, that certain *erythemata* of children and fat subjects, the *itch, herpes labialis,* under many circumstances, and *zona* itself, constitute merely local diseases, and merely require local treatment, although some of these affections do occasionally manifest themselves as the crisis, or the consequence, of a generally disordered state. On the other hand, the *eczema,* the *impetigo* and the *pseudo-tineæ* of infants at the breast, or during dentition, appear to be purifying or counteracting maladies, dependant upon certain general conditions which ought to be taken into consideration. Plethora, either local or general, a gritty and roughened state of the primæ viæ, nervous and circulatory fluxions, dependant on the revolutions of age, and many other circumstances well known to the practitioner, give rise to the *exanthemata,* to *acne* or *acne rosacea,* and to various species of cutaneous affections, the cause of which is elsewhere than in the seat of the apparent evil. Lymphatic debility is favorable to the development of *favus,* or the true *tinea;* the scrofulous diathesis often produces *lupus* or *herpes exedens;* the syphilitic virus regulates and has under its dependance special forms of cutaneous disease. It is very evident then that the local ought never to be considered independently of the general state, in the study of this branch of pathology. When we observe also the spontaneous development of a very large number of *dartrous* affections, the hereditary appearance of some, the resistance which they offer to the most ably directed treatment, the facility, and I may say, the obstinacy, with which they reproduce themselves, the often dangerous effects consequent upon their suppression, &c., it appears difficult to entirely reject this ancient opinion, which has become proverbial, not only with the faculty, but with the uninitiated, as to the existence of an internal cause, a peculiar diathesis, which produces and keeps up, in many cases, the diseases of the skin." P. 15.

Among the *occasional causes* may be mentioned 1, *hereditary causes;* thus the disposition to *dartrous* affections seems transmissible, and M. Gibert states in a note that he has had "opportunities of proving in many subjects the hereditary nature of *icthyosis, psoriasis* or *lepra, impetigo, eczema,*

and *lupus.* 2. A few diseases, as *itch,* and perhaps *tinea* (the author includes the various forms of porrigo under this term), are *contagious* ; but there is every reason to believe the various dartrous diseases are not so ; and we must take care not to permit the occurrence of mere coincidence, arising from similar regimen, habits, and constitution to deceive us. 3. *Anatomical* and *physiological causes.*—A fine delicate skin easily penetrated by the blood is very liable to these affections ; and a rough, thick, oily skin, which performs its functions with difficulty, and occurring chiefly in bilious temperaments, is also subject to papular and other eruptions. Alibert remarks that certain temperaments are especially liable to certain forms of the disease ; " thus, lymphatic and lymphatico-sanguineous persons are principally liable to scaly and furfuraceous eruptions ; sanguineous and sanguineo-lymphatic persons are frequently tainted with the impetigo of Willan, and bilious and melancholic subjects are particularly liable to pustular and squamous affections." The influence of *age* is often very marked ; as is seen in the eruptions of the head and face of children, the pustules on the forehead at puberty, the obstinate cutaneous disease at the critical period of life of women, and those which result from the imperfect cutaneous action of old age. 4. *Hygienic Causes.*—To this head must be referred the effects of *climate* and of season. The want of *cleanliness* is one of the most potent of all causes. The influence of bad and insufficient *diet* has always been recognized. The suppression of perspiration, even when only local, often gives rise to cutaneous disease. " We have often seen the sudden suppression of the customary perspiration in some part of the body, the feet for example, followed by dartrous eruptions on the ears and in other places." The suppression of the intestinal flux, and of the menstrual and hæmorrhoidal discharges also give rise to various cutaneous diseases. Masturbation exerts a marked effect in inducing certain pustular affections, and prolonged continence produces often the form of acne known as the " pimples of chastity." Frank states that eunuchs enjoy a singular immunity from skin disease. Certain occupations produce certain forms of disease, *e. g.* prurigo of beggars, the papular affection called " grocers' itch," the psoriasis of washerwomen, &c. Some occupations, although unfavorable to health, are yet liable to few cutaneous diseases, as those of nightmen, miners, &c. Sedentary professions and studious habits, especially if an exciting regimen is maintained, often produce pustular or squamous eruptions or *prurigo* of the genitals. Powerful or long-continued depressing emotions frequently induce skin-disease. 5. *Pathological Causes.*—Thus scrofula and syphilis are frequently the source of these affections. Gout and rheumatism sometimes favour their development or alternate with them ; and the cutaneous diseases not unfrequently induce each other. " But a practical remark, to which our predecessors attached a high importance, and which we ought not to neglect here, notwithstanding the oblivion into which it appears to have fallen, is, the intimate connexion which exists, in many cases, between the internal organs and the tegumentary affections, and the danger which we run in too suddenly suppressing, or even sometimes in treating too methodically, in some persons, the cutaneous maladies to which they are subject."

*Progress.*—This is usually slow, and accompanied by exacerbations frequently without obvious cause. The tendency of these affections to creep from one part to the other has given rise to the Greek term *herpes* and the Latin *serpigo*.

"It is not very uncommon to see them terminate by *metastasis;* either their suppression involves the affection of another organ than the skin, or this affection itself, pre-existing, becomes very intense, and causes the dispersion of the cutaneous malady; or lastly, in some cases, we must admit the transportation of this species of movable principle, which our predecessors did not hesitate to designate, correctly or incorrectly, under the title of dartrous. The greatest number of acute and chronic diseases is observed to be consequent on the disappearance of eruptions; whether they may or may not be dependant upon this suppression, it is not always so easy to establish in practice as one might at first imagine. Nevertheless we may truly affirm that examples of *mental alienation, epilepsy, arachnitis,* internal inflammations, and various organic lesions, occurring more or less quickly after the sudden cessation of cutaneous maladies, ought to render the physician extremely prudent in their treatment when they appear constitutional, that is to say, dependant upon a general and well-characterized modification, either of such and such a viscera in particular, or of the entire economy.

"Sometimes a kind of *crisis* is observed in these diseases. In some cases we see a fever, an internal inflammation, a sanguineous or mucous flux (particularly if it has been anteriorly suppressed) cause, by its sudden appearance, the cessation of the pre-existing cutaneous affection. Occasionally too we observe the manifestation of an exanthema, or a dartrous eruption, or the return of a previously suppressed cutaneous malady, bring about the crisis of an internal inflammation. '*Alvi affectiones, ut diarrhea, colica,* &c., *in cutis affectiones migrant,*' says Baglivi. We know the success obtained by some practitioners from the inoculation of the *itch,* the employment of sudorifics, the application of irritating ointments or vesicatories, in cases where internal diseases, more or less severe, succeeded to cutaneous maladies. M. Alibert alludes to a severe affection of the air-passages caused by the driving in of a dartrous eruption after a cold bath, and which yielded to the return of this eruption, induced by the use of diaphoretics, and the application of a large blister upon the chest." P. 24.

*Treatment.*—M. Gibert observes, that the Greek and Arabic writers laid great stress upon the employment of *hygienic* means; and adds, "we must not here omit to notice, that the cleanliness, the regular life, the change of habits, which the class of people who are received into hospitals undergo, are often sufficient of themselves to dissipate the cutaneous diseases, which have procured their admission, and that, if we do not attach sufficient importance to this favorable influence, we may be led to attribute too much to the pretended efficacy of remedies, which have had little or no part in effecting a cure."

The ancients, too, and even the practitioners of last century, much insisted upon a methodical preparation of the patient by bleeding, baths, purging, &c. prior to commencing the special treatment of the skin-disease.

"In the present day we have, for the most part, fallen into the contrary extreme. Practitioners of the highest celebrity do not hesitate to attack immediately, upon first sight, with caustic, or other substances, the most inveterate cutaneous affections, without even having recourse to any internal remedy during the whole course of treatment.

"There are evidently two dangers to avoid; and if it is absurd to follow and observe indiscriminately, in all cases and with all subjects, the therapeutic precepts which the ancients practised, with more discernment perhaps than we give them credit for, it is not more rational to entirely neglect this preparatory method, and particularly all general treatment, in diseases which frequently depend upon a particular constitutional state, and the cure or speedy disappearance of which is not always so exempt from dangers as some physicians of *the new school* affect to believe." P. 29.

The author, after enumerating at length the various medicinal substances which have at various periods been employed for the relief of cutaneous diseases, lays down some general rules for guiding the practitioner through what he well terms the "therapeutic labyrinth"—leaving the exceptions to be indicated when treating of the individual diseases. "It is particularly in the diseases which we are now considering, that the application of energetic agents, advised by different authors, requires all the tact and acumen of an experienced physician. Here, as in many other cases, the most efficacious remedy may become a poison in the hands of ignorance; poison itself can become a salutary remedy, when it is suitably administered by a clever practitioner."

1st. *Antiphlogistics.*—These are called for in their various degrees, from depletion to a low and soothing regimen, especially at the commencement of treatment in vigorous subjects, when much irritation prevails, and the progress of the disease is rapid.

"Nevertheless, we must mention, beforehand, that whilst twenty years ago, *specific* methods were abused, the sulphureous and other excitants in usage being applied off-hand and indiscriminately to everything which bore the name of *dartre;* in the present day, on the contrary, prepossessed with the modern idea, which tends to attribute to inflammation every shade of alteration which the tissues, and the skin amongst them, can present, we insist more and more frequently upon antiphlogistics, and particularly upon topical emollients, which, in many cases, have merely the effect of prolonging the evil, by favouring the fluxion and morbid exhalation which may be noticed in the integuments in most dartrous diseases." P. 36.

2nd. *Astringents.*—Although these often possess great advantages over antiphlogistics, considerable caution is necessary in their employment. "It is not uncommon to see pruriginous, vesicular, and humid eruptions suppressed at their very commencement, without any danger or inconvenience, by applications of burnt meal, *extract of lead,* ointments with *white lead,* or even by *ice, cold-water, red-wine,* &c. How often has *eczema* (*dartres squameuses humides*), which continues to spread and extend itself under the influence of emollients, been rapidly cured by tonics, astringents, and discutients. However, they are generally only recommended at rather an advanced period in the treatment. M. Bland, of Beaucaire, has recently called the attention of practitioners to the advantages of *soot* in the treatment of dartrous affections, particularly favus, lupus, and acne rosacea. (Revue Med. T. 2, 1834.)"

3rd. *Narcotics,* such as *henbane, belladonna, opium,* &c. are sometimes resorted to, externally or internally, when there is violent or insupportable itching: but these are only palliative, and by their repelling power may occasionally become injurious.

4th. "Caustic applications, such as *the nitrates of silver and mercury,* are frequently employed, in the present day, for the dispersion of dartrous maladies, even to the entire neglect of all internal remedies. This does not appear to us a very rational method, and we think that these energetic agents ought to be reserved for three principal circumstances: 1st, when a cutaneous, and particularly a contagious, disease, such as the itch, is at its commencement, and we can hope by this means to arrest it in its birth; 2nd, when an inveterate cutaneous disease has been already combated by internal and external means of a different kind, and we have no reason to fear the effects of the suppression of the local evil upon the economy, nor the continuance of the general morbid disposition which has already produced it, and will produce it again: 3rd, when it becomes necessary to arrest the progress of *dartre rongeante* or lupus." P. 38.

5th. *Issues.*—These are so abundantly employed in French practice in cases which English practitioners would be content with the use of purgatives, that the abominable odour they produce may be detected in every public vehicle, and in all other assemblages of persons, even of the young. In skin diseases, however, they are now prohibited by many practitioners, as tending to maintain an undue tegumentary irritation and fluxion—itself predisposing to the return of these affections. Blisters or cauteries are useful towards the termination of tedious cases, especially if any internal organ seems to be suffering much; and it is in the class of cases in which the suppression of long-continued eruptions has given rise to internal disease, that these and other revulsive measures are useful.

6th. *Tonics.*—These are very useful in scrofulous and lymphatic subjects; and the *alkaline subcarbonates* frequently form admirable combinations. The *muriates of lime* and *barytes* are sometimes useful in scrofulous disease, cautiously given in small doses.

7th. *Purgatives.*—The ancients used these much in skin affections, and some modern physicians declare they obtain nearly constant success by their exclusive employment. Most chronic cutaneous affections are accompanied by constipation.

8th. *Sudorifics.*—The various descriptions of baths are of powerful agency in cleansing the skin from squamæ and crusts, and re-establishing its functions, always perverted in chronic affections. An artificial *sea-water* bath may be made by adding six or eight pounds of common salt, rendering it less irritating by the addition also of half to a pound of gelatine. *Alkaline* baths may be formed by four ozs. of subcarb. potass and some gelatine, and M. Gibert often employs a *saponaceous* bath, formed by dissolving half-a-pound of soap in a strong decoction of bran and then adding it to the water. The *sulphureous* baths may be formed by adding four ozs. of the solid, or eight ozs. of the liquid sulphuret of potass. The crystallised hydro-sulphate of soda, however, furnishes a preparation for baths less odoriferous and more approaching those of nature. Sudorific drinks, as sarsaparilla, guaiacum, mezereon, to which antimonial preparations are added, are especially useful in inveterate syphilitic eruption.

9th. *Specifics.*—Although a large class of skin diseases may be amenable to these, it by no means follows that they should always be employed to the exclusion of all other medication. Most of the vegetable juices, baths, &c. mentioned by the author, do not seem to us to deserve the title of specifics. Sulphur for the itch is about our only one; although the various other stimuli enumerated often prove curative in the different chronic diseases.

M. Gibert thus concludes his general remarks upon the treatment.

"In order to render more precise the general rules which we have laid down, we will take a particular example, and will mention the order to be observed in a methodical and rigorous treatment.

"Supposing a patient, with a very strong and vigorous constitution, to be affected with an *impetigo :* we should commence by prescribing a preparatory treatment (low diet, bleeding, baths, diluents, purgatives). Afterwards, in the course of the first period, relaxing drinks, an abstemious and regular regimen, and, occasionally, a warm bath ; still later, bitter and purifying drinks, and vapour baths ; lastly, sulphur baths, preparations of sulphur externally and internally, topical astringents, corrosives, and even caustics, a blister, too, if the eruption were intractable and circumscribed, (the crusts being previously detached by emollient applications). In following this course, we effect a slow, it is true, but a certain cure, with but little probability of a relapse.

"It is here that we are reminded of the old adage, so full of useful application, either in medicine or surgery :

"Sat citò si sat benè." P. 41.

The author, not embracing in the present work the consideration of the more general affections of the system manifesting themselves upon the surface, as scarlatina, variola, rubeola, erysipelas, &c. presents us with the following modification of Willan's Classification.

1. *Exanthemata,* containing three species, *Urticaria, Roseola, Erythema.*
2. *Bullæ,* containing *Pemphigus* and *Rupia.*
3. *Vesiculæ,* containing three, *Scabies, Herpes, Eczema.*
4. *Pustulæ,* containing *Ecthyma, Acne, Impetigo, Tinea* (the *Porrigo* of Willan ; the affections to which the term tinea is ordinarily applied being termed by M. Gibert *Pseudo-tineæ.*)
5. *Papulæ,* containing *Prurigo, Lichen.*
6. *Squamæ,* containing, *Icthyosis, Pityriasis* and *Lepra* ; (Dr. Gibert considering Psoriasis and Lepra to be the same affection.)
7. *Tubercula,* containing *Lupus, Elephantiasis Græcorum* and *Arabica,* and *Cheloidea.*
8. *Maculæ,* containing *Ephelides, Nævus,* and *Purpura.*

The omission of so many important diseases very much diminishes the utility of the work for others than such as are attending the practice of his hospital.

Under each of the above orders of cutaneous diseases is embraced the consideration of the modifications produced in the various species by syphilis ; and, at the end of the work, we are presented with a "*General Summary of the History of Syphilitic Eruptions.*" This account of the "*Syphilides*" we are disposed to consider the best portion of the work.

M. Gibert prefaces his observations by some remarks on primary symptoms, which are not without interest.

"The *primary venereal chancre* is one of the most common and characteristic phenomena of syphilis. Nevertheless, the mode of formation of this ulcer has been generally badly described in the special treatises on venereal diseases ; there is even a writer who has been anxious to found a *new* therapeutic method on an error which has crept into the description which many praiseworthy authors have given of it. Incorrectly believing that a *vesicle* commonly preceded the ulceration, this physician recommended the application of the *ectrotic* method (cauterization) in the treatment of chancres at their commencement, as a means calculated to destroy the evil in its embryo, and prevent the absorption of the virus and the development of consecutive phenomena, without the necessity of resorting to internal treatment. But, besides cauterization being a means commonly employed for a very long time, and its effects as a preservative often having failed, observation proves that the venereal chancre does not ordinarily commence by a *vesicle;* nothing has been perceived but a little swelling and redness, and soon afterwards an ulceration, which has formed without any cuticular elevation. It is very important to recollect here the characters of a *vesicular* cutaneous affection, of short duration, which sometimes occupies the internal surface of the prepuce, and which may be followed by excoriations, which have been mistaken for chancres. This slight disease, which we have described under the name of *herpes præputialis,* is characterised by small vesicular groups, commonly accompanied by redness and itching: they most frequently dry up in a week, are not irritated by improper applications, or other stimulating causes. The venereal chancre properly so called, can only assume temporarily, and in some exceptional cases, that vesicular or (pustular) appearance at its commencement, which we have already mentioned with regard to the flat tubercle. Like this latter, in fact, the chancre appears to have its seat in the tegumentary follicles; and the follicular inflammation may accidentally present at its commencement this vesicular appearance, but not from a *vesicle* or a *pustule,* properly so called. It is only in *inoculation* made with the lancet, that veritable cutaneous pustules are developed, and afterwards give rise to a chancre.

"Some physicians are determined to look upon *buboes* as nothing but an engorgement of the lymphatic glands, caused by the irritation of the parts where the absorbent vessels which lead to them rise; and, consequently, are unwilling to admit the existence of buboes *at the first onset.* We have many times observed this sort of bubo (without any other anterior symptom) in men and women. Even more, we have *inoculated* in these cases the pus of the bubo, and caused the formation of the characteristic pustule to which the venereal chancre succeeded. This inoculation has been always successful with us when we have waited till the wound formed by the spontaneous or artificial opening of the suppurated bubo has been converted into a veritable venereal ulcer, which generally happens in a few days.

"The *flat* moist *tubercles,* incorrectly described under the name of flat *pustules,* are a very common primary phenomenon. They show themselves on the scrotum, the vulva, and in the neighbourhood of the anus, usually without any other local symptoms preceding their appearance.

"It is very generally agreed, in the present day, that *gonorrhœa* may depend on various causes, and that it is not always syphilitic. But, on the other hand, we know, without doubt, that constitutional syphilis very frequently succeeds to this primary symptom; and that, unfortunately, we are in possession of no certain sign by which we can distinguish the venereal gonorrhœa from that which merely consists in simple inflammation of the urethra.

"M. Ricord pretends that the only blenorrhagiæ susceptible of giving rise to consecutive symptoms, are those which are accompanied by a urethral chancre. But this is a supposition which does not appear to us sufficiently justified by the few exceptional cases in which a venereal ulcer has been discovered deep in the urethra affected with this discharge. Time and direct observation have done justice to the old opinion, which regarded as *ulcerated* the mucous membranes affected with puriform catarrh: relative to gonorrhœa in particular, autopsy has many times revealed that the urethral canal followed in this respect the common law. It is a question which I shall only just allude to here, and which will be found treated of more fully in my *Manuel des Maladies Vénériennes.* Inoculation will be, according to M. Ricord, a certain means of deciding this point. But as in cases of this kind it usually only gives rise to negative results, I do not think that we can induce from it the future innocuity of blenorrhagia unsuccessfully inoculated. In fact, we have not hitherto succeeded in communicating by inoculation any but one venereal symptom (chancre); and yet, not only the *flat tubercles* and *vegetations* are transmissible from individual to individual, but even certain consecutive symptoms appear in some cases to be able to transmit themselves by contagion, although no one has ever been able to inoculate them with the lancet." P. 341.

All the elementary forms of cutaneous disease may be produced by syphilis, so that we have an *exanthematous* syphilide, a *pustular*, a *papular*, and so on. They are distinguished, however, from analagous forms of the diseases, arising from common causes, by various marks, sufficiently satisfactory.

"The *coppery* discolouration is one of the most certain; it may be found in all the forms of cutaneous syphilis, although it may not be always equally apparent, equally easy to seize upon, especially to an inexperienced eye. The *ulceration* which succeeds to many species, has also so characteristic an appearance, that it is impossible to mistake its nature. Those deep, round, hard-edged, callous, and perpendicularly cut excavations; those *serpiginous* ulcerations, which form segments of a circle and of spiral lines; those thickened, greenish, darkish *crusts*, deep in the dermis, which sometimes cover the ulcerations, and which particularly succeed the syphilitic bullæ, pustules, and tubercles, have evidently no deceptive appearance. The *cicatrices* themselves, unequal, spiral, or rounded, white and depressed, which follow ulcerated tubercles and pustules, have characters by which their origin can be recognised. Moreover, in the greatest number of cases, past circumstances, the pre-existence of primary local phenomena, the co-existence of other venereal symptoms, such as discharges, flat tubercles on the genitals or anus, *granulated erosions* of the uterine neck, ulcers of the velum palati, exostoses, &c., assist in removing all doubts which we may entertain." P. 342.

*Syphilitic roseola* is by far the most common form of the *exanthematous* syphilides, M. Gibert having only twice seen examples of syphilitic *urticaria.* The roseola often shows itself during the primary symptoms, as gonorrhœa or chancre, but, as the other forms, for the most part as a consequence of ill-treated or neglected venereal disease. "More frequently than the other forms, we see it suddenly break out in persons, who at a more or less distant period have been tainted with syphilis, owing to some moral or physical disturbance which seemed to determine the manifestation of a poison, the germ of which had long been latent in the system." The spots of syphilitic differ from those of ordinary roseola in their copper colour, their permanence, number, which is greater, and duration. Their true character may be masked during the acute stage of the first few days, but it then becomes revealed by the small, dull, greyish, copper-coloured, or livid stains, which are left for a long period.

Of the *Bullous Syphilide* there is only one species, *syphilitic rupia*, which is very common, occurring generally in old and intractable cases and in cachectic habits, and co-existing for the most part with other secondary symptoms, as iritis, periostitis, &c. "The concomitant signs, the copper-coloured or livid areola which surrounds the bullæ, the thick greenish crusts which they form on drying, the deep subjacent ulceration, with perpendicular edges and a greyish base, and the white, depressed cicatrices which they afterwards leave, serve to distinguish the *syphilitic*

from the *simple,* and even the *prominent* rupia, with which it has more analogy.

The *Vesicular Syphilide* is very rare, and is situated on the limbs and trunk, and not the organs of generation. In only one instance has M. Gibert observed the disease taking on the form of *eczema.* In the other instances it has resembled *varicella,* consisting then of large, isolated vesicles, having a slow march and dull appearance, and being sometimes surrounded by a small copper-coloured areola.

Of *Pustular Syphilide* there exists two varieties; the one, resembling the pustules of *ecthyma* or even of *variola,* the other those of *acne.* Even experienced observers have at first sight mistaken the marks of variola for traces of syphilitic pustules, or the elevations of variola in the primary stage for the commencement of a syphilitic ecthyma.

"The following are the distinctive characters of the *pustular syphilide,* whether it shows itself in the form of *phlyzacious* pustules (*syphilitic ecthyma*), or those called *psydracious,* and which present some analogy to the small, pointed, and isolated pustules of the genus *acne.*

"In the first case, the pustules, generally more voluminous than those of *ecthyma vulgare,* are frequently situated on the face, sometimes on the limbs, sometimes also on the trunk; they slowly and imperfectly mature, are covered with thickened brownish crusts, under which ulcerations often form, which leave behind them round, white, and depressed cicatrices. This variety of pustular syphilide is very rare, if it is not quite among the newly-created ones.

"In the second case, which is much more common, the pustules are numerous and approximated, very frequently extending over the whole tegumentary surface (except the face and the forehead, where they are extremely rare), sometimes confined to the trunk or limbs; they ripen imperfectly, quickly dry up without leaving any sensible crust, leaving behind them livid, copper-coloured, or greyish marks, which last a very long time; or even small superficial cicatrices, round, and of a whitish colour." P. 198.

*Papular Syphilide* is very common, and shows itself under two varieties. In the first, there are slightly voluminous papules, always however more voluminous than those of *non-syphilitic lichen,* their colours, slow progress, great number, the greyish spots they leave behind, and the absence of itching, distinguishing them from lichen and prurigo. In the other variety, the prominences are larger, flattened, and covered with small *squamæ,* bearing much resemblance to the squamous syphilide. This syphilide is sometimes observed during the existence of chancre or blenorrhagia, when these have continued for a long space of time.

"We never see, as in the ordinary *lichen,* merely a region of slight extent remain affected, or very marked intervals separate the papules, as in *prurigo;* but, on the contrary, the skin is dry, coppery, violet-coloured, sprinkled with prominent papules, or marked with small, greyish, or livid spots, so that we can scarcely find here and there—on the back, for example, or on both surfaces of the arms—any points where the integuments have preserved their colour and their integrity. The face alone is very often untouched; still the forehead is nearly always affected. The papules, after having lasted some time, terminate by resolution, and are re-placed by others, which are renewed, and keep up the eruption for weeks, months, and even years. This form of the syphilitic eruptions, very common in the adult, is never observed in infancy. At least, we have never met with it at this age. On the contrary, as we have already had occasion to remark, the *pustular* syphilides (with large pustules), and the *tubercular* (with *flat* tubercles), are common in this latter period. Although less severe than the two forms which we have just mentioned (since it does not involve ulceration), the *papular* syphilide frequently is of a long duration, and offers an obstinate resistance to remedial measures." P. 224.

*Squamous Syphilide* is one of the most common forms of syphilitic eruption; and is distinguished from the genus *lepra* (under which term M. Gibert includes psoriasis) by the following characters.

"The colour of the patches is more obscure, livid, or coppery; they are covered with small greyish scales, very different from the shining and pearly ones of *psoriasis;* rarely confluent, they are commonly of a lenticular form, which more resembles *psoriasis guttata* than any other eruption. It sometimes, however, disposes itself in rings more or less analogous to those of *lepra vulgaris.* M. Biett is even of opinion that the scaly eruption which the English authors have described under the name of *lepra nigricans,* is syphilitic.

"When the patches of *psoriasis* are deprived of their squamæ, when the chronic march of the disease, the bilious tint of the subject, and the slight habitual colouring of the integuments tend to obscure their commonly rosy colour, the diagnosis becomes less easy. If, however, we carefully weigh the past and concomitant signs, especially if we seek in the places which it selects (the elbows and knees in particular), the characteristics, generally more marked than elsewhere, of the eruption, it is very rare that we can mistake for syphilitic, an affection which often deceives the inexperienced and inattentive." P. 259.

M. Biett considers a peculiar eruption of the palm of the hand somewhat resembling *psoriasis palmaria,* to belong to the scaly syphilides, and terms it *syphilide cornée.* Small scaly prominences are observed with hard centres, analagous to the *corns* of the feet. The diagnosis is sometimes much assisted by the co-existence of copper-coloured scaly patches on the wrist.

The *Tubercular Syphilide,* which has often been miscalled pustular, is the most common form of cutaneous syphilis. The author enumerates five principal varieties. 1. *Flat* tubercles found about the genitals and anus, or between the toes. 2. *Round* and larger tubercles, sometimes acquiring the size of a nut, often remaining indurated without ulcerating, and appearing in small numbers about the head, face, and neck. 3. A *granulated* tubercle, having its seat at the angles of the mouth or alæ nasi, being "small, greyish or copper-coloured, and occasionally presenting small cracks or superficial ulcerations." 4. The *serpiginous tubercle,* occurring on the trunk and furrowing "with crustaceous and serpiginous ulcerations, which form segments of a circle, or irregular rings, and species of well-characterized letters or figures." 5. *Herpetiform tubercles,* much resembling the preceding, appearing especially on the forehead and hairy scalp, and characterized by small rings composed of flattened and closely approximated tubercles, which ulcerate superficially, and are covered with small adherent crusts. Besides the above well-marked varieties, there are other tubercular eruptions which cover the limbs, back and chest, continuing indurated, or terminating in resolution; sometimes ulcerating and encrusting, and at others, imperfectly suppurating.

"It is most frequently the *tubercular* form which the syphilitic eruptions which we see happen in *nurses* who are infected by a child, assume. Small tubercular elevations form close to the nipple, and are converted into crustaceous ulcerations; flat tubercles afterwards show themselves on the anus and the parts of generation: sometimes there is a general eruption. Nearly always, if the evil is not arrested in time, other consecutive symptoms supervene, such as ulcers in the glands of the neck, granulated erosion of the neck of the uterus, &c. A very remarkable thing, and one which we have often proved at the Hôpital de Lourcine, is, that when the nurse suckles her own child at the same time, the latter may remain well if the breast which it sucks is so; whilst the breast which is devoted to the infected nursing becomes alone affected, although there may already have supervened in the nurse the various consecutive symptoms which we have alluded to. Nevertheless, if the suckling were prolonged without the nurse undergoing any treatment, there is no doubt that the second child would, after a certain time, contract the disease.—(See my *Manuel des Maladies Vénériennes.*)" P. 320.

*Maculæ Syphiliticæ.*—These are commonly consecutive of some of the syphilides we have enumerated and are distinguished from *ephelides* and *pityriasis versicolor* " in that they are commonly of a round shape, rarely exceed the size of a half-crown, are, in general, less numerous, are met with on the face, and particularly on the forehead and eyebrows. They are most frequently of a coppery-red, sometimes almost blackish, give rise to slight or no itching, and are only rarely the seat of a trifling desquamation." They are, nearly always, too accompanied by other secondary symptoms. It is still undetermined whether these stains are ever elemental, Biett regarding them as mere traces of pre-existent forms. In two instances, however, M. Gibert believes he has seen such.

In the *treatment* of the syphilides, M. Gibert speaks highly of the *cinnabar fumigations*, either partial or general. In other cases he has used, with good effect, the new combinations of *mercury* with *iodine*, *cyanogen* and *bromine* in the form of *frictions* to the diseased parts. But as these preparations are very active and irritating, they require great care for their employment. In a letter addressed to the translator he speaks highly of the syrup of the *deuto-ioduret* of *iodine*. The dose is from 1 to 2 tea-spoons full daily, each of which contain $\frac{1}{5}$ grain of the bi-iodide of mercury, and 10 grains of the iodide of potassium. " I attribute the use of iodide of potassium in large doses, become so general in the treatment of constitutional syphilis, to the success which I obtained by means of this syrup at the Hôpital de Lourcine in 1836." M. Biett entertains a high opinion of the treatment of constitutional syphilis by *corrosive sublimate* upon Dzondi's method; *i. e.* commencing with $\frac{1}{20}$ gr. and gradually augmenting it to one or two grains in the 24 hours. A grain of the sublimate is divided into 24 pills, one of which is at first given daily, adding another every two or three days until 24, 30, or 36 are taken in divided doses daily. In almost desperate cases this plan has admirably succeeded. The danger of such a medicine administered without due precaution is obvious. When mercury has not been deemed admissible, the *muriate of gold*, applied by frictions to the gums in doses of $\frac{1}{4}$-$\frac{1}{8}$ gr., mixed with inert powder, has been used with marked success in some cases, though little confidence is in general placed in its efficacy. *Sudorifics* and *Narcotics* are often valuable means.

Simple, alkaline, or sulphur baths, combined with the internal use of corrosive sublimate or the proto-ioduret of mercury, form the ordinary treatment at St. Louis. If the disease is very obstinate, Dr. Scatigna's plan of employing mercurial friction is resorted to, viz. the deposition of a drachm of the ung. hyd. in the axilla daily for three days, when the patient takes a bath, and the same treatment is re-commenced.

Upon *Syphilis in the Infant*, we find the following observation.

"It is always by a syphilitic eruption that the poison transmitted from the mother is discovered in a new-born infant. It usually shows itself towards the close of the first, or at the beginning of the second, month after birth. It has its seat on the perineum, the internal surface of the thighs, and the neighbourhood of the organs of generation, in the form of *flat tubercles,* or syphilitic *ecthyma,* and from thence it spreads over a variable extent of the integuments. A little later, the mucous membranes become affected, particularly the mouth, and the labial commissures; it is then that, if the child is confided to a nurse to suckle, the nipple of the latter ulcerates, and the disease is communicated thereby. Syphilis, in the new-born infant, is always a severe malady; it frequently falls a victim to it in a few weeks. Nevertheless, if it is well constituted, if we at once treat the nurse and the child, and if they are both placed in favourable hygienic conditions, a cure is very easily obtained. This is much surer too in cases where the infant is merely secondarily infected, that is to say, if it has received the poison posterior to birth, as, for example, in the very common case where the nurse has suckled her own child conjointly with the strange one infected by its parents.

"We generally confine ourselves, in children, to the employment of topical applications, such as unctions with the following pommade:

℞ Opiate Cerate . . . . . . . . . ℥j.

Ammoniacal Oxychloride of Mercury . ʒj.

Care is, moreover, taken to prescribe emollient baths, and to watch that the infant is properly cleansed. If it is being suckled, the nurse is made to take corrosive sublimate internally, in the form of pills, such for instance as the following:

℞ Extract of Aconite . . . . . gr. xij.
Powdered Opium . . . . . . . gr. ij.
Deuto-chloride of Mercury . . . gr. ij.

Mix and divide into eight pills; one to be taken every morning at breakfast." P. 357.

This, which we consider the best portion of the work (for we cannot allow the justice of the Translator's eulogium on the chapter on Elephantiasis), has occupied so much of our space, that we have only room for a few observations on Scabies.

*Contagion of Scabies.*—"Scabies is an accidental and contagious disease, which is propagated by the mediate or immediate contact of affected with sound individuals. But this propagation is impeded or favoured by a number of accessory circumstances, and there is nothing so common as to see persons, and particularly medical men, expose themselves in a more or less direct manner to the contagion, without becoming tainted with the malady. It is thus that in hospitals it is rare to see the servants of the establishment, the pupils, or the physicians contract it, although there are daily intimate and close communications between them and the patients, and they generally take but little precaution in examining, visiting, and touching those who are affected with the disease. We would not, however, wish this observation to inspire, either professional or non-professional persons, with too great a security: for, on the other hand, we now and then see examples of its development, for the first time, in individuals who for some time past have been in circumstances favourable to its contraction, without being able to give a reason for the innocuity in the one case, the unfortunate result in the other, of the relations which have not appeared to change their nature.

"The most numerous examples of the itch are to be met with in young persons; and this is owing to the greater number which this age, compared to others, presents, and to the occupations, and the frequenting places of debauch, so peculiar to this period of life.

"Women are much less tainted with it than men, which, doubtless, may be attributed, in a great measure, to the kind of life they lead." P. 97.

*Diagnosis.*—Although this is easy in the simple form of the disease, it becomes somewhat difficult when it is complicated by other diseases, as eczema, &c., modifying its characters. Even its simple form is often confounded by medical men with lichen or prurigo; and great inconvenience to the patient and loss of reputation to the practitioner may be the result. In *prurigo* there are no vesicles but only papules, which preserve the natural colour of the skin, or are covered by little dark crusts caused by their having been excoriated by the patient's nails. The papules are found especially upon the back, shoulders, and the dorsal and external aspect of the limbs; the itch vesicles exhibiting themselves between the fingers, on the wrists, the articular folds, and on the internal aspect of the limbs, the accompanying itching being much less painful than that of prurigo. The sensation from scratching is rather agreeable, while in prurigo the skin may be lacerated, and yet relief not obtained. *Lichen* is also a papular affection, and bears no resemblance to scabies, except in the lichenoid affection termed *grocer's itch.* In this the papules are grouped on the dorsum of the hand, the itch vesicles preferring the inter-digital spaces. The vesicles of *eczema rubrum* are more closely grouped and more inflamed than those of itch: they are especially found in the axillæ, the ears, forehead, and genital organs; and give rise to smarting rather than itching. Unfortunately they may complicate scabies. *Ecthyma vulgare* gives rise to pustules, which are rarely numerous, and not to vesicles, and is not attended with decided itching. They are usually converted into isolated crusts.

*Treatment.*—The author believes that, in some cases, sulphur should be given internally as well as employed externally; *e. g.* when the cure is desired to be rapid, the inconvenience of suppressing a long-continued eruption is dreaded, or its character is not very clearly manifested. The sulphur fumigations are not easy of application save in hospitals, and are not even speedy means of cure there. The lotion proposed by Dupuytren, and modified by Alibert, is a good one. In one bottle ℥j. of sulphuret of potash or soda is dissolved in 2lb. of water; and in another, there are ℥ij. of sulphuric acid. At the time of application, the patient places a glass of each in a bason filled with hot water, and washes the part affected for half an hour morning and evening. This remedy has only a slight odour, and does not stain the linen: but M. Gibert prefers the *hydro-sulphate of crystallized* soda or Anglada Salt. The *liniment of Jadelot* (sulphuret of potash ℥ij., white soap ℔j., oil of poppies ℔ij., ol. thyme ʒj.) often proves irritating, giving rise to eczema rubrum. *Valentin's* liniment is composed of ℥j. ol. amygd., ʒj. sulphuret, and ℈j. of camphor, is less irritating, and much preferable; but, even with sulphur baths, it requires 10 to 15 days to cure the disease by its means. But we cannot understand the author as respects the time required, for he states, the sulphur ointment requires also 15 days to effect a cure—an infinitely longer period than is ever, according to our own experience, necessary for the use of this nastiest but best of preparations. The progress of the disease may be arrested if the patient is seen early enough, by opening the first vesicles that appear, and cauterizing with the *nitrate of silver.* Friction with common olive oil is efficacious in children.

